# Additions to the review of Chinese *Enochrus*, with description of a new species (Coleoptera, Hydrophilidae, Enochrinae)

**DOI:** 10.3897/zookeys.480.8898

**Published:** 2015-02-02

**Authors:** Fenglong Jia, Renchao Lin

**Affiliations:** 1 Institute of Entomology, Sun Yat-sen University, Guangzhou, 510275, Guangdong, China; 2 School of Life Science, Sun Yat-sen University, Guangzhou, 510275, Guangdong, China

**Keywords:** Coleoptera, Hydrophilidae, Enochrinae, *Enochrus*, aquatic beetles, new species, Palearctic Region, Oriental Region, China

## Abstract

A new species, Enochrus (Methydrus) limbourgi **sp. n.**, is described from Jiangxi Province, Southeast China, and illustrated. Subgenus *Enochrus* s. str. Thomson, 1859 is recorded for the first time in China, based on the record of Enochrus (s. str.) melanocephalus (Olivier, 1792) from Inner Mongolia. The male of Enochrus (Hydatotrephis) liangi Jia & Zhao, 2007 is described for the first time.

## Introduction

Jia and Wang (2010) revised all species of *Enochrus* known from China except *Enochrus hybridus* Hebauer, 2005. Of six subgenera of *Enochrus* Thomson, 1859, four have been reported from China: *Methydrus* Rey, 1885, *Holcophilydrus* Kniz, 1911, *Hydatotrephis* MacLeay, 1871, and *Lumetus* Zaitzev, 1908 (Hansen 1999, Jia and Zhao 2007, Short and Hebauer 2006, Short and Fikáček 2011). However, the subgenus *Enochrus* Thomson, 1859 which is reported from Nearctic, Afrotropical, Oriental and Palearctic Regions, has not been recorded to China up to now. In 2013, *Enochrus algarum* Jia & Short, 2013 was described from Eastern China. The species was not assigned to any subgenus because of a few special characters that do not match any known subgenus. In total, 21 species of *Enochrus* have now been reported from China.

Enochrus (Hydatotrephis) liangi Jia & Zhao, 2007, the sole species of the subgenus occurring in China, was described based on a single female (holotype) from Guizhou Province, China (Jia and Zhao 2007) and it was redescribed in English in detail in 2010 based on the same specimen (Jia and Wang 2010). Unfortunately, male has remained unknown.

In 2014, we have examined some new freshly collected material of *Enochrus* from various parts of China. Among those, we discovered a male belonging to yet undescribed species from Jiangxi Province, a series of *Enochrus liangi* including males, and a male specimen of *Enochrus* s. str. from Inner Mongolia. All these new findings are summarized in this paper, including the description of the new species. Based on the results, we may confirm that all subgenera of *Enochrus* except *Hugoscottia* Knish, 1922 (endemic to the Neotropical Region and not likely to be discovered in China) do occur in China.

## Material and methods

The holotype and some of the paratypes were dissected and the male genitalia mounted in glycerine on transparent plastic labels attached below each specimen. Specimens and genitalia were examined with the use of an Olympus SZX10 compound microscope. Habitus photographs were taken with ZEISS Axio Cam HRC, Discovery V20. Aedeagus photographs were taken with an Axioskop 40. The photographs were stacked using Auto-Montage software.

Morphological terminology follows Hansen (1991) and Komarek and Beutel (2007) for general morphology. Classification follows Short and Fikáček (2013).

Examined specimens are deposited in the following collections:

NMPC National Museum, Prague, Czech Republic;

SYSU Collection of Sun Yat-sun University, Guangzhou, China.

## Taxonomy

### Enochrus (Hydatotrephis) liangi

Taxon classificationAnimaliaColeopteraHydrophilidae

Jia & Zhao, 2007

Figs 1 , 2 , 6–11 , 18

Enochrus (Hydatotrephis) liangi Jia and Zhao 2007: 252.

#### Type material.

**Holotype:** female (SYSU): CHINA: Guizhou: Leishan County: Fangxiang: female, 14. ix. 2005, Coll. Shuang Zhao.

#### Additional material examined.

18 males and 14 females (NMPC, SYSU): China, Jiangxi Province, Jing’an county, Daqishan forest farm, ca. 28.67°N, 115.07°E, 350 m a.s.l., in a natural pool, 18.vii.2014, Renchao Lin leg [with Chinese and English labels]; 1 female (SYSU): China, Sichuan Province, Leshan, Emeishan, Qingyinge, 29°34'N, 103°07'E, 750 m, 7.vi.2014, Renchao Lin lgt.

#### Diagnosis.

**Figures 1–11.**
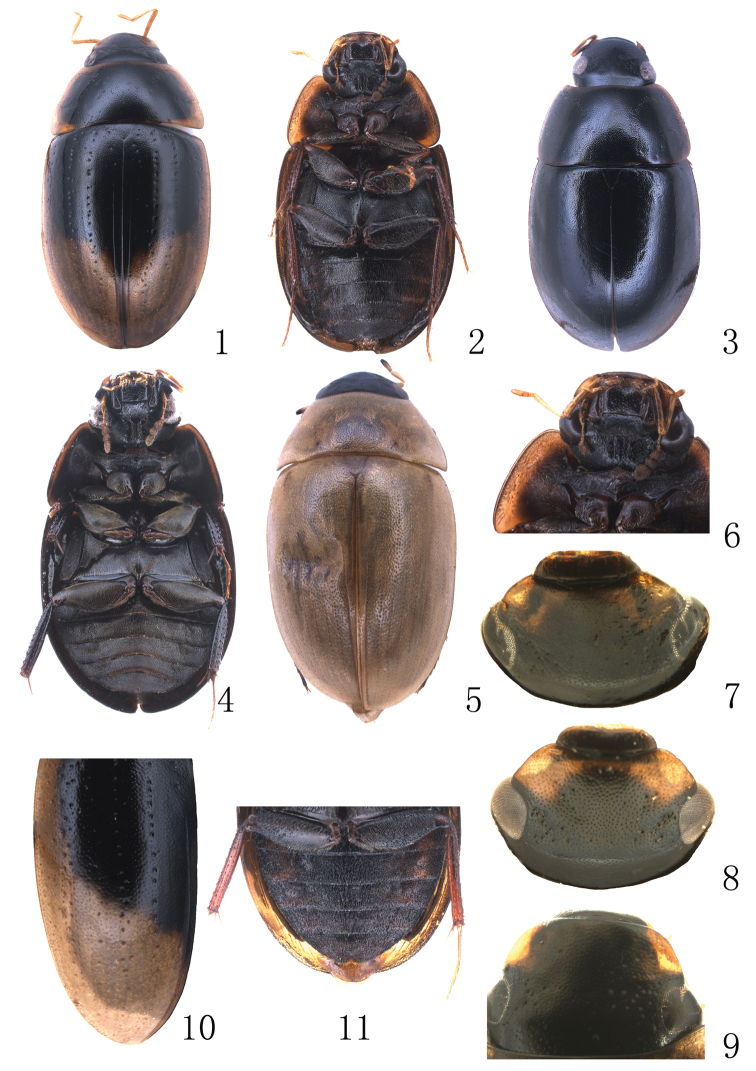
**1–5** Habitus **1–2** *Enochrus liangi*: **1** dorsal view **2** ventral view **3–4** *Enochrus limbourgi*: **3** dorsal view **4** ventral view **5** *Enochrus melanocephalus* **6–11** *Enochrus liangi*: **6** head, ventral view **7–9** head, dorsal view **10** elytron (left) **11** abdomen.

Head with large preocular spots, reaching frontoclypeal suture posteriorly and reaching inner margin of eyes inwards (Fig. 9), sometimes each preocular spot separated into two spots by a black patch (Fig. 7), or preocular spots connected medially (Fig. 8). Elytron black with posterior half yellow, and yellow colour extends anteriorly as broad band along the lateral margin (Figs 1, 10). Maxillary palps yellow, not dark apically, subequal to the width of head anterior to eyes (Fig. 6). Elytron with a short series of punctures in front of sutural stria, which are same size as other serial punctures (Fig. 10). Fifth abdominal ventrite with apical emargination fringed with stiff yellowish setae (Fig. 11). Aedeagus similar to Enochrus (Methydrus) japonicus (Sharp, 1873) (Fig. 19), with parameres curved outwards apically, median lobe narrow with sharp apex (Fig. 18). The colour is very similar to Enochrus (Holcophilydrus) laoticus Hebauer, 2005 and the latter also with parameres curved outwards subapically. However, *Enochrus laoticus* Hebauer with 10 elytral series of punctures; mesosternal process conical, not depressed laterally; median lobe of aedeagus broader and with small emargination apically, as long as parameres.

**Figures 12–21.**
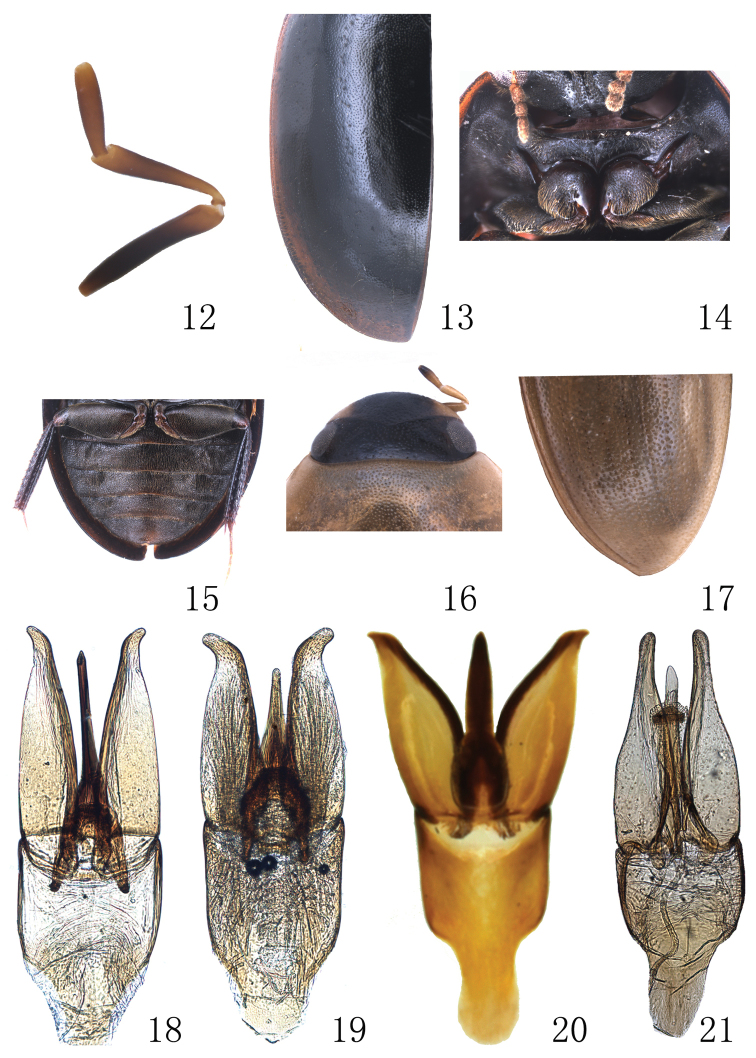
**12–15** *Enochrus limbourgi*: **12** maxillary palpomeres **13** elytron (left) **14** prosternum **15** metalegs and abdomen **16–17** *Enochrus melanocephalus*: **16** head **17** posterior portion of elytron **18–21** Aedeagi: **18** *Enochrus liangi* **19** *Enochrus japonicus* **20** *Enochrus limbourgi* **21** *Enochrus melanocephalus*.

#### Description of male.

External morphology identical with female. Male genitalia: phallobase slightly longer than parameres. Parameres broad, longer than median lobe, obliquely truncated inwards and strongly bent outwards apically. Median lobe narrower than parameres, gradually narrowed from base to apex, sharp apically (Fig. 18).

#### Distribution.

China (Guizhou, Sichuan, Jiangxi). New for Sichuan and Jiangxi.

#### Habitat.

The series from Jiangxi was collected at margin of natural pools with mud sediment and sparse grass. Lots of *Enochrus japonicus* (Sharp) were also collected in the same pool.

### Enochrus (Methydrus) limbourgi sp. n.

Taxon classificationAnimaliaColeopteraHydrophilidae

http://zoobank.org/ED3AC1DF-A67A-42DA-9834-C9D4D6FE4271

Figs 3–4 , 12–15 , 20

#### Type material.

**Holotype:** male (SYSU): China, Jiangxi Province, Jing’an county, Zaodu town, Nanshan village, 29.01°N, 115.16°E, 315m, 19.vii.2014, light trap, Ren-Chao Lin leg.

#### Diagnosis.

Size 7.3 mm. Head without preocular spots (Fig. 3). Second maxillary palpomere pronouncedly and extensively darkened except extremity, apical palpomere yellowish brown, not dark apically (Fig. 12). Prosternum with a low carina medially (Fig. 14). Head, pronotum and elytron with fine and very dense punctures (Fig. 13). Scutellum with a few coarse and strong punctures. Claws of anterior and middle legs in male distinctly strongly and angularly curved, bearing a smaller basal tooth. Fifth abdominal ventrite with apical emargination fringed with stiff yellowish setae (Fig. 15). Aedeagus with parameres curved outwards apically. Median lobe oval broadened basally, abruptly narrowed ca. half, apical half much narrower than parameres, sharp apically (Fig. 20)

The new species is most similar to Enochrus (Methydrus) eubenangeei Watts, 1998 which is endemic species to Australia in its large size, black color, punctures on dorsal surface, mesoclaws thickened basally and bent in male, median lobe of aedeagus oval, broadened basally, parameres obliquely truncate apically. In contrast to *Enochrus eubenangeei* Watts, *Enochrus limbourgi* is characterized by: pronotum and elytron with distinct pale yellow-brown margin; prosternum with a low distinct median carina; elytra with five series of punctures; claws of male middle tarsi bent, broadly thickened basally; aedeagus with parameres broad, obliquely truncate apically and weakly curved outwards subapically (parameres similar to Enochrus (Methydrus) aliciae Watts, 1998 in form).

The size of this species is the largest in the known species of Enochrus (Methydrus) from the Oriental and southern Palearctic Regions. The median lobe of aedeagus is clearly different from other known Asian species. It is very easy to distinguish this species from other species in Asia by size, punctures and aedeagus. Compared with Chinese species, it is closed to *Enochrus japonicus* Sharp by size and colour. It can easily be distinguished from the latter by pronotum and elytra with denser ground punctures, serial punctures on elytra less strong and less coarse, mesosternal process strongly depressed laterally, median lobe of aedeagus oval broadened basally, parameres sharp apically.

#### Description.

***Form and Colour*.** Body length 7.3 mm, body width 4.0 mm. Body oval, moderately convex. Dorsum of head, pronotum and elytron black, with lateral margins of pronotum and elytron distinctly yellow-brown. Antennae yellow-brown with club black. Maxillary palps with second maxillary palpomere pronouncedly and extensively darkened except extremity (Fig. 12); third palpomere slightly darkened medially, apical palpomere yellowish brown, not dark apically (Fig. 12). Labial palps yellow, not darkened apically. Venter, including legs, black, tarsomeres yellow-brown.

***Head.*** Antennae with scape ca. as long as antennomeres 2–3 combined. Maxillary palps subequal to the width of head anterior to eyes; apical palpomere about three-quarters of penultimate in length (Fig. 12). Anterior margin of clypeus straight medially. Labrum with a median row of setiferous systematic punctures, distinctly coarser than the surrounding ground punctation. Frons and clypeus with ground punctation dense and coarse, distance between ground punctures 1.0–1.2× the width of one puncture; setiferous systematic punctures well pronounced, ca. 3× as large as ground punctures. Mentum subquadrate, with anterior margin slightly depressed medially, not emarginate anteriorly (Fig. 4), ground punctures moderately coarse.

***Thorax.*** Ground punctation on pronotum and elytron similar to that on head, distance between ground punctures 1.0–1.2× the width of one puncture. Elytron with five rows of serial punctures (including lateral series) clearly larger than surrounding ground punctation; without short series of punctures in front of sutural stria, the third series with a few punctures that are distant between punctures. Sutural stria present in posterior half of elytra. Prosternum not tectiform, with a low distinct median carina (Fig. 14) and a transverse groove behind anterior margin. Mesoventrite with a median process which is strongly impressed laterally and with a backwardly pointing projection, rising to level of mesocoxae, apex of the projection with a few long setae. Metaventrite with a very indistinct elongate oval glabrous area posteromedially, longer than wide; glabrous area slightly more than half the total length of the metaventrite. Mesofemora densely pubescent except in apical fifth (Fig. 15). Metafemora with pubescence as in mesofemora (Fig. 15). Posterior tarsomeres with a fringe of long swimming-hairs dorsally. Anterior claws in male strongly and angularly curved, bearing smaller basal tooth; claws of male middle tarsi of similar shape as those of anterior tarsi, but slightly weakly angularly curved; posterior claws only slightly curved, without basal tooth.

**Abdomen.** Ventrites uniformly and densely pubescent. Fifth (apical) abdominal ventrite with apical emargination fringed with stiff yellowish setae (Fig. 15).

**Aedeagus.** Phallobase about 1.2× as long as parameres. Parameres broad, longer than median lobe, abruptly truncate and bent outwards apically. Median lobe oval broadened basally, abruptly narrowed ca. half, apical half much narrower than parameres, sharp apically (Fig. 20).

#### Etymology.

The specific name is after Dr. Pol Limbourg, an entomologist in Intitute Royal des Sciences Naturelles, Brussels, Belgium, who helped us a lot when senior author studied types of *Enochrus* in d’Orchymont’s collection in Brussels.

#### Distribution.

China (Jiangxi), known only from the type locality.

#### Habitat.

The holotype was collected by light trap.

### New faunistic record

#### Enochrus (s. str.) melanocephalus

Taxon classificationAnimaliaColeopteraHydrophilidae

(Olivier, 1792)

Figs 5 , 16–17 , 21

##### Material examined.

1 male (SYSU): CHINA: Inner Mongolia, Huangqihai wetland, in a rain pool, 20.vi.2013, Li Shi leg.

##### Distribution.

This is a widespread species in Europe. It is distributed “from France and the British Isles to Asia Minor” (Hansen 1987). It is only known in Algeria (Hansen, 1999) in Africa and in Israel and Uzbekistan in Asia (Hansen 1999; Hendrich and Hendrich 2005). It is very possible that this species should be distributed in Mongolia and Middle Asia. **New for China.**

### Update of the key to Chinese *Enochrus*

The key to the species of genus *Enochrus* of China published by Jia and Wang (2010) should be modified as follows, in order to include *Enochrus limbourgi* sp. n. and *Enochrus melanocephalus* recorded as new to China here, and *Enochrus algarum* described by Jia and Short (2013):

**Table:** 

1	Elytra with 10 striae or regular series of punctures.	1a
–	Elytra without striae except for a sutural stria, but often with 3 distinct rows of punctations and lateral series of punctures	3
1a	Maxillary palps very short, half as long as the width of the head. Elytra with ten rows of weakly impressed striae. Fifth abdominal ventrite entire, without any emargination, truncation, or thickened setae	***Enochrus algarum* Jia & Short, 2013**
–	Maxillary palps almost as long as or longer than the width of the head. Elytra with ten very strongly impressed series of punctures or continuous striae. Fifth abdominal ventrite with emargination apically, with a tuft of golden setae (subgenus *Holcophilydrus*)	**2**

3	Mesoventral elevation conical, not compressed from the sides, strongly vertically declining posteriorly towards middle coxae (Fig. 9) (subgen. *Hydatotrephis*); posterior margin of pronotum finely emarginate; elytra black with posterior half yellow brown. Median lobe of eadeagus very narrow and sharp apically, parameres broad, curved outwards subapically	***Enochrus liangi* Jia & Zhao, 2007**
–	Mesoventral elevation more or less strongly keel-shaped, compressed from the sides, or only gently declining posteriorly between middle coxae	**3a**
3a	Apical palpomere of maxillary palp as long as the third palpomere; elytra without setiferous systematic punctures; fifth (apical) abdominal ventrite without apical emargination (subgenus *Enochrus* s.str.)	***Enochrus melanocephalus* (Oliver, 1792)**
–	Apical palpomere of maxillary palp distinctly shorter than the third palpomere; elytra with distinct setiferous systematic punctures, or fifth abdominal ventrite with apical emargination	**4**

14	Body large (5.7–7.3 mm), dorsal coloration black. Parameres widened apically and strongly curve outwards	**14a**
–	Body smaller (2.3–4.8 mm), at least yellowish brown on elytra, Parameres not widened apically and not strongly curved outwards	**15**
14a	Size 7.3 mm. Elytral serial punctures less coarse and strong, diameter of elytral serial punctures about 2–3× as wide as ground punctures; sutural stria present in posterior half of elytral length. Mesoventral elevation strongly impressed laterally; Median lobe of aedeagus oval broadened basally	***Enochrus limbourgi* sp. n.**
–	Size 5.7–6.0 mm. Elytral serial punctures coarser and stronger, diameter of elytral serial punctures about 4–5× as wide as ground punctures; sutural stria reaching basal fourth of elytral length. Mesoventral elevation conical, directed somewhat posteriad, not strongly compressed from the sides	***Enochrus japonicus* (Sharp, 1873)**

## Supplementary Material

XML Treatment for Enochrus (Hydatotrephis) liangi

XML Treatment for Enochrus (Methydrus) limbourgi

XML Treatment for Enochrus (s. str.) melanocephalus

## References

[B1] Hansen M (1987) The Hydrophiloidea (Coleoptera) of Fennoscandia and Denmark. Fauna Entomologica Scandinavica 18: 1–254.

[B2] Hansen M (1991) The Hydrophiloid Beetles. Phylogeny, Classification and a Revision of the Genera (Coleoptera, Hydrophiloidea) Biologiske Skrifter, Det Kongelige Danske Viedenskabernes Selskab 40: 1–368.

[B3] Hansen M (1999) World Catalogue of Insects 2: Hydrophiloidea (s.str.) (Coleoptera). Apollo Books, Stenstrup, 416 pp.

[B4] Hendrich L Hendrich E (2005) A contribution to the knowledge of the water beetle fauna of Uzbekistan (Coleoptera:Hydradephaga, Hydrophiloidea, Staphylinoidea and Dryopoidea). Linzer Biologische Beiträge 37(1): 425–434.

[B5] Jia F-L Zhao Sh (2007) Hydrochidae and Hydrophilidae. In: Li Z Yang M Jin D (Eds) Insects from Leigongshan landscape. Guizhou Science and Technology Publishing House, Guiyang, 251–255 [in Chinese, English abstract]

[B6] Jia F-L Wang Y (2010) A revision of the species of Enochrus (Coleoptera: Hydrophilidae) from China. Oriental Insects 44: 361–385. doi: 10.1080/00305316.2010.10417622

[B7] Jia F-L Short AEZ (2013) *Enochrus algarum* sp. n., a new hygropetric water scavenger beetle from China (Coleoptera: Hydrophilidae: Enochrinae). Acta Entomologica Musei Nationalis Pragae 53(2): 609–614.

[B8] Komarek A Beutel R (2007) Phylogenetic analysis of Anacaenini (Coleoptera: Hydrophilidae: Hydrophilinae) based on morphological characters of adults. Systematic Entomology 32: 205–226. doi: 10.1111/j.1365-3113.2006.00359.x

[B9] Short AEZ Hebauer F (2006) World Catalogue of Hydrophiloidea – Additions and Corrections, 1. (1999–2005) (Coleoptera). Koleopterologische Rundschau 76: 315–395.

[B10] Short AEZ Fikáček M (2011) World catalogue of the Hydrophiloidea (Coleoptera): additions and corrections II (2006–2010). Acta Entomologica Musei Nationalis Pragae 51(1): 83–122.

[B11] Short AEZ Fikáček M (2013) Molecular phylogeny, evolution and lassification of the Hydrophilidae (Coleoptera). Systematic Entomology 38: 723–752. doi: 10.1111/syen.12024

[B12] Watts CHS (1998) Revision of Australian *Enochrus* Thomson (Coleoptera: Hydrophilidae). Records of the South Australian Museum 30(2): 137–156.

